# On Resection of the Stomach

**Published:** 1882-10

**Authors:** 


### On Resection of the Stomach.

When Péan published first his observations on resection of the pylorus, four years ago, he was accused of making vivisections on the human person. But shortly afterward Billroth, at Vienna, studied with his pupils the conditions and means of excuting this operation. After so many ovariotomies, laparotomies, gastrotomies and resections of the intestines had been successfully performed, it was but a question of time to perform the resection of the pylorus. Merrem, in the year 1810, originated the idea of performing extirpation of the pylorus; Guenther practiced it on three dogs. Gussenbauer and Winiwarter demonstrated afterward the possibility of gastrotomy on dogs. Kayser and Wehr repeated the experiments, and studied the resection of the stomach. We will give the experiences of those operators in the following lines: "The conclusions arrived at are as follows: 1. It is absolutely necessary to multiply the sutures in order to prevent the escape of fluids into the peritoneal cavity. To reunite an intestine which has a diameter of two centimeters on the cut surface, from 24 to 30 sutures must be applied. 2. The hæmorrhages are mostly due to the omentum. The dissection ought to be made very slowly, and in short strokes, taking care to secure the divided parts in two ligatures. 3. The unsuccessful cases are due to imperfections in the operation. The peritoneum should not be the cause of death. It is necessary to examine the patient under anæsthesia before the operation, to ascertain the extent of the tumor, its configuration and its adhesions. Billroth has demonstrated that it is not advisable to operate when the neoplasm adheres to the pancreas, to the descendant portion of the duodenum and to the anterior abdominal glands. It is necessary to wait in case the patient be jaundiced. Great dilatation of the stomach will indicate a fatal result. The patient must become accustomed to the stomach pump. The stomach must be emptied before the operation with the stomach pump, and antiseptic surgery should be applied. The incision is made in the linea alba above the tumor (Billroth), and it should be from eight to twelve centimeters long. Hemostasis should be complete before the peritoneum is opened. To explore the stomach is usually difficult, but it presents itself in the aperture in cases of great dilatation, and then it is drawn forward. Some surgeons propose to reunite the opened stomach with provisional sutures, in order to make the operation extra-peritoneal. If there should be too many or too intimate adhesions, it is better to close the wound without any further interference. To isolate the tumor from the great and small omentum, it is necessary to make a number of double ligatures, dividing between the ligatures every time. The resection of the tumor should thus be without any loss of blood, and warm towels should cover all the neighboring parts. After having isolated all parts which have to be elevated, a sponge is laid under these parts, to absorb all the fluids. To prevent this escape of fluids some operators have compressed with hard rubber pincettes; others apply ligatures. Rydygier uses elastic compressors, which he places one centim. above the tumor. These compressors consist of two pieces of iron thirteen to fifteen centim. long, and three-fourths centim. thick, and they are covered with rubber. One is placed on the anterior part of the stomach, and the other on the posterior part. They are fastened by pieces of rubber. The incision is made slowly, with scissors, into the stomachal wall, an assistant ligaturing the bleeding vessels and sponging the parts with phenic or carbolic acid. It is very difficult to obtain two apertures which will fit exactly. Rydygier makes an oblique excision on the duodenum, and he secures thus two surfaces of equal extent. Billroth unites a part of the stomachal orifice with sutures until the other part corresponds with that of the duodenum. The margins of the duodenum are united to those of the stomach in the following manner: The sutures are introduced into the mucous membrane of the stomach; they penetrate the muscular wall and make their exit through the serous membrane one centim. from the surface of the incision; they then enter the serosa of the duodenum, and pass through the muscularis and mucosa of the same organ. A double line of sutures is made by most operators, in the space of two to three millims. from each other, to prevent the escape of fluid into the peritoneal cavity, and some sutures are introduced on the posterior aspect to adapt the mucous membrane. Catgut and carbolized silk have both been applied with success, but silk is preferable. The reunion of the abdominal walls is made as in ovariotomy. In the first days after, the patient is nourished by the rectum. Twenty-seven resections of the pylorus have been performed, with six recoveries. Shock and prolapsus are the most prominent causes of death. The prolonged application of chloroform causes a weakness of the heart, with following death. When there are adhesions to the pancreas, Billroth closes the wound without extirpating the pylorus. Among 903 cancers of the stomach, 542 were limited to the pylorus. At all events, the extirpation of the pylorus will always be a difficult and dangerous operation. —*Archives Générales de Médecine.*

